# Dietary Nitrate: Ward et al. Respond

**Published:** 2006-08

**Authors:** Mary H. Ward, Theo M. de Kok, Patrick Levallois, Jean Brender, Gabriel Gulis, James VanDerslice, Bernard T. Nolan

**Affiliations:** Division of Cancer Epidemiology and Genetics, National Cancer Institute, National Institutes of Health, Department of Health Human Services, Bethesda, Maryland, E-mail: wardm@mail.nih.gov; Department of Health Risk Analysis and Toxicology, University of Maastricht, Maastricht, the Netherlands; Institut National de Santé Publique, du Québec and Unité de recherche en santé publique, Centre Hospitalier Universitaire de Québec, Québec, Canada; Texas A & M School of Rural Public Health, College Station, Texas; Southern Denmark University, Odense, Denmark; Washington State Department of Health, Olympia, Washington; U.S. Geological Survey, Reston, Virginia

We read with interest the letter by L’hirondel et al. regarding our workgroup report (Ward et al. 2005). L’hirondel et al. describe the research on methemoglobinemia, cancer, adverse reproductive, and other health outcomes as “extensive” and state that the range of results found is what would be expected if there were no correlation between these health outcomes and drinking water nitrate exposure. We disagree with their assessment of the literature. The etiologies of specific cancers and adverse reproductive outcomes are likely to be different from each other, and there are too few well-designed studies of any particular health outcome to draw conclusions about risk.

L’hirondel et al. correctly point out that nitrate levels are higher in certain vegetables than in most drinking water sources. Indeed, when nitrate levels are below the regulatory limit of 10 mg/L nitrate-nitrogen (nitrate-N), the majority of nitrate intake comes from vegetables (Chilvers et al.1984; Levallois et al. 2000). Ingested nitrate from diet and drinking water is secreted at high concentrations by the salivary glands and is reduced to nitrite by bacteria in the mouth. In the acidic stomach, the nitrite is rapidly converted to nitrous acid and then to nitric oxide and nitrosating species, which can react with amines and amides to form *N*-nitroso compounds (NOC), the potential causative agents in the etiology of specific cancers, adverse reproductive outcomes, and diabetes. Low gastric nitrite concentrations, as reported by Vu et al. (1994) and McColl (2005), do not mean that nitrite is not involved in endogenous nitrosation, as implied by L’hirondel et al.

Human studies have shown that water nitrate exposure above the regulatory limit increases urinary excretion of NOC (Mirvish et al. 1992; Moller et al. 1989; Vermeer et al. 1998). NOC formation also increased after a meal of vegetables high in nitrate and low in ascorbic acid (e.g. beets, celery); however, NOC formation was inhibited after a meal of these vegetables together with vegetables and fruits containing ascorbic acid and nitrate (Knight et al. 1991). Numerous studies have shown that the formation of NOC in the stomach is inhibited by dietary antioxidants found in vegetables and fruits (Bartsch et al. 1988; Mirvish et al. 1998; Vermeer et al. 1999). Therefore, inhibition of endogenous NOC formation may account for some of the observed inverse associations between vegetable intake and many cancers and adverse reproductive outcomes.

To adequately evaluate the risk associated with consumption of nitrate in drinking water at the regulatory limit of 10 mg/L nitrate-N [background levels are typically < 1 mg/L (Nolan and Hitt 2003)], studies must account for the potentially different effects of dietary and water sources of nitrate. Well-designed studies include the assessment of exposure for individuals (e.g., case–control, cohort studies) in a time frame relevant to disease development, and the evaluation of factors affecting nitrosation. Estimating NOC formation via nitrate ingestion requires information on diet and drinking water nitrate, inhibitors of nitrosation (e.g., vitamin C, polyphenols), nitrosation precursors (e.g., red meat, nitrosatable drugs), and medical conditions that may increase nitrosation (e.g., inflammatory bowel disease).

Only a few such studies evaluated risk among potentially susceptible groups (reviewed by Ward et al. 2005), and two studies found significantly elevated risks associated with water nitrate levels below the regulatory limit (Brender et al. 2004; De Roos et al. 2003). Higher nitrate levels in drinking water were associated with an increased risk of colon cancer among individuals with high red meat or low vitamin C intakes (De Roos et al. 2003). Higher water nitrate ingestion was linked with neural tube defects in the offspring of women who used nitrosatable drugs during the peri-conceptional period (Brender et al. 2004).

We agree with L’hirondel et al. that diarrhea, in addition to high water nitrate exposure, can cause methemoglobinemia in infants; in our article (Ward et al. 2005) we stressed the need for further studies to clarify the role of drinking water nitrate exposure. Nevertheless, it is important to note that the regulatory limit does not include a safety factor; rather, it is based on available data supporting no observed adverse effect for methemoglobinemia in infants (the most sensitive subpopulation) [U.S. Environmental Protection Agency (EPA) 1991]. Therefore, we do not agree that the regulatory limit is overprotective as suggested by L’hirondel et al.

Until more well-designed studies are conducted and evaluated, we reject the conclusions by L’hirondel et al. that enough evidence has been gathered to safely raise the drinking water limit for nitrate. Raising the regulatory limit, and thereby allowing the increased intake of drinking water nitrate, would likely result in increased exposure to endogenously formed potentially carcinogenic and neurotoxic *N*-nitroso compounds and possibly result in new cases of methemoglobinemia.
